# Transient expression of βC1 protein differentially regulates host genes related to stress response, chloroplast and mitochondrial functions

**DOI:** 10.1186/1743-422X-7-373

**Published:** 2010-12-30

**Authors:** Saiqa Andleeb, Imran Amin, Aftab Bashir, Rob W Briddon, Shahid Mansoor

**Affiliations:** 1 Agricultural Biotechnology Division, National Institute for Biotechnology and Genetic Engineering, Faisalabad, Pakistan

## Abstract

**Background:**

Geminiviruses are emerging plant pathogens that infect a wide variety of crops including cotton, cassava, vegetables, ornamental plants and cereals. The geminivirus disease complex consists of monopartite begomoviruses that require betasatellites for the expression of disease symptoms. These complexes are widespread throughout the Old World and cause economically important diseases on several crops. A single protein encoded by betasatellites, termed βC1, is a suppressor of gene silencing, inducer of disease symptoms and is possibly involved in virus movement. Studies of the interaction of βC1 with hosts can provide useful insight into virus-host interactions and aid in the development of novel control strategies. We have used the differential display technique to isolate host genes which are differentially regulated upon transient expression of the βC1 protein of chili leaf curl betasatellite (ChLCB) in *Nicotiana tabacum*.

**Results:**

Through differential display analysis, eight genes were isolated from *Nicotiana tabacum*, at two and four days after infitration with βC1 of ChLCB, expressed under the control of the *Cauliflower mosaic virus *35S promoter. Cloning and sequence analysis of differentially amplified products suggested that these genes were involved in ATP synthesis, and acted as electron carriers for respiration and photosynthesis processes. These differentially expressed genes (DEGs) play an important role in plant growth and development, cell protection, defence processes, replication mechanisms and detoxification responses. Kegg orthology based annotation system analysis of these DEGs demonstrated that one of the genes, coding for polynucleotide nucleotidyl transferase, is involved in purine and pyrimidine metabolic pathways and is an RNA binding protein which is involved in RNA degradation.

**Conclusion:**

βC1 differentially regulated genes are mostly involved in chloroplast and mitochondrial functions. βC1 also increases the expression of those genes which are involved in purine and pyrimidine metabolism. This information gives a new insight into the interaction of βC1 with the host and can be used to understand host-virus interactions in follow-up studies.

## Background

Geminiviruses are economically important plant pathogens and are characterized by twinned isometric particles containing single-stranded (ss)DNA genomes of 2.5-3.0 kb [1] that replicate through double-stranded (ds)DNA intermediates by a rolling-circle mechanism [2]. The family *Geminiviridae *is divided into four genera, (*Begomovirus*, *Mastrevirus*, *Curtovirus *and *Topocuvirus*) that encompass viruses that differ in genome organization as well as their insect vectors. Begomoviruses are transmitted by the whitefly *Bemisia tabaci *and have either monopartite or bipartite genomes. Monopartite begomoviruses are often associated with circular, ssDNA satellites that are collectively referred to as betasatellites (formerly known as DNA β). Betasatellites have recently been found to be associated with some bipartite begomoviruses and are required by some of their helper begomoviruses to induce *bona fide *disease symptoms in plants. Numerous economically important diseases and even the earliest recorded plant viral disease are now known to be caused by begomovirus/betasatellite complexes [3,4].

Betasatellites are widespread in the Old World, where monopartite begomoviruses are known to occur. Numerous distinct betasatellites, from various economically important hosts and diverse locations, have been cloned and have been found in most cases to contribute significantly to disease symptoms [5]. Analysis of betasatellite sequences reveals a highly conserved organization consisting of an adenine-rich region and a region of sequence highly conserved between all betasatellites (known as the satellite conserved region [SCR]). The SCR contains a potential hairpin structure with the loop sequence TAA/GTATTAC that has similarity to the origins of replication of geminiviruses and nanoviruses. Betasatellites encode only a single gene, known as the bC1, located on the complementary-sense strand, is conserved in position and size in all betasatellites [6,7].

Chilli leaf curl betasatellite (ChLCB) is associated with chilli leaf curl disease (ChLCuD), a significant constrain to chilli production across the Indian subcontinent [8,9]. Saeed et al. [5] demonstrated that tobacco plants transformed with the βC1 of Cotton leaf curl Multan betasatellite (CLCuMB) under the control of the *Cauliflower mosaic virus *35S promoter, or with a dimer of CLCuMB, exhibited severe disease-like phenotypes, while plants transformed with a mutated version of the βC1 appeared normal. Qazi et al. [10] showed that expression of CLCuMB βC1 from a *Potato virus X *vector induced symptoms typical of cotton leaf curl disease (CLCuD) in the absence of the helper begomovirus. These results demonstrated that CLCuMB βC1 is the major determinant of symptoms of the CLCuD complex [10].

The interactions between plants and viruses are complex and involve several types of responses that may or may not cause disease in the host [11]. In compatible interactions, the invading virus is able to infect and replicate within a susceptible plant to cause disease. Alternatively, the host may trigger innate immunity mechanisms that restrict virus movement and prevent disease onset. In both situations, viral pathogens severely disturb plant growth and development, due to their effect on cellular metabolism [11]. Viral infection produces a plethora of symptoms derived from biochemical and metabolic changes in cells, tissues and even in the whole plants which are susceptible and hypersensitive resistant hosts. Huang et al. [12] and Sui et al. [13] demonstrated that plant viruses cause severe impact on host gene expression and protein activity due to the activation of a set of genes and the inactivation of others. The gene expression profile in the host plant changes according to the timing and localization of the infection, as the virus spreads from cell to cell away from the site of inoculation [14,15].

The present studies are aimed at identifying host genes and pathways that are induced by ChLCB βC1. This may be achieved using differential RNA display technology. This technique is based on "differential display reverse transcriptase polymerase chain reaction" (DDRT-PCR), first described by Liang and Pardee [16]. This method has the advantage of technical simplicity, a lower bias against rare messages and a requirement of only small quantities of starting mRNA. Several modifications of the original technique have been reported with some solutions to the key problems identified by some authors [17]. Stress responses have been studied using DDRT-PCR in *C. elegans *and *S. cerevisiae *[18-20]. DDRT-PCR has been applied in many laboratories to identify genes involved in signal cascades.

The identification of host genes affected by ChLCB βC1 may provide useful insights into virus-host interactions and provide targets for novel control strategies. By differential display analysis we have identified *N. tabacum *genes differentially regulated in response to the transient expression of ChLCB βC1 protein. Subsequently the effects of βC1 expression on each gene identified were verified by quantitative real time PCR analysis.

## Results and Discussion

**Figure 1:**
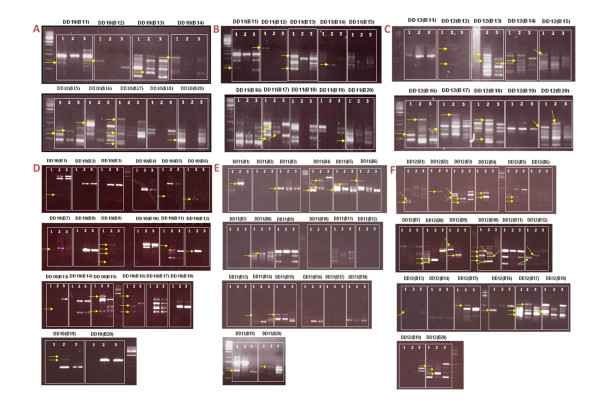
**A. Identification of genes differentially expressed in response to βC1 by differential display analysis at two and four days after infiltration**. In each combination of arbitrary and anchored primers, lane 1 represents 100 ng/μl of cDNA (pSAβC1pGreen0029), lane 2 shows 10 ng/μl of cDNA (pSAβC1pGreen0029) and lane 3 indicates 100 ng/μl pGreen0029 in *Agrobacterium tumefaciens *strain GV 3101. Differential display analysis two days after inoculation with DD10 (B11-B20); B. DD analysis two days after inoculation with DD11 (B11-B20); C. DD analysis after two days of inoculation with DD12 (B11-B20); D. DD analysis after four days of inoculation with DD10 (B1-B20); E. DD analysis four days after inoculation with DD11 (B1-B20); F. DD analysis four days after inoculation with DD12 (B1-B20). The bands eluted for analysis are indicated (→).

We have made a further modification of the DDRT-PCR technique by utilizing the mRNA fraction instead of total RNA and by resolving the products of DDRT-PCR on 1% agarose gels stained with ethidium bromide [16]. We have identified several genes which were differentially expressed at 2 dpi and 4 dpi. Two different concentrations of cDNA (100 ng/μl and 10 ng/μl; Figures 1A-F) were used, of which ninety seven differentially expressed genes (DEGs) were amplified by different anchored and arbitrary primer pairs (Table 1; Figures 1A-F). The anchored and arbitrary are random decamer primers, and used as reverse and forward primer for cDNA synthesis. Agroinfiltration was used for transient expression of βC1 (ChLCB) under 35S promoter. DDRT-PCR showed different bands of transcripts in comparison to control plants. Some of the primer combinations did not yield an amplification product (Figures 1A-F). At 2 dpi no difference was observed in control and infected plants as indicated in DD10 (B7, B18); DD11 (B15, B16, B19); and DD12 (B11, B19), respectively (Figures 1A-C). On the other hand, at 4 dpi same pattern was also observed in DD10 (B2, B3, B4, B8, B10, B14, B17, B18, B20), DD11 (B2, B6, B7, B9, B10, B11, B12, B13, B16, B17, B18), and DD12 (B6, B7, B11, B14, B15) respectively (Figures 1D-F).

**Table 1:** Sequences of oligonucleotide primers used in the study

Use	Primer	Sequence
	Anchored Primer	

	DD10	5'-TTTTTTTTTTTG-3'
cDNA	DD11	5'-TTTTTTTTTTTC-3'
	DD12	5'-TTTTTTTTTTTA-3'

	Arbitrary Primer	

	B-01	5'-GTTTCGCTCC-3'
	B-02	5'-TGATCCCTGG-3'
	B-03	5'-CATCCCCCTG-3'
	B-04	5'-GGACTGGAGT-3'
	B-05	5'-TGCGCCCTTC-3'
	B-06	5'-TGCTCTGCCC-3'
	B-07	5'-GGTGACGCAG-3'
	B-08	5'-GTCCACACGG-3'
	B-09	5'-TGGGGGACTC-3'
DDRT-PCR	B-10	5'-CTGCTGGGAC-3'
	B-11	5'-GTAGACCCGT-3'
	B-12	5'-CCTTGACGCA-3'
	B-13	5'-TTCCCCCGCT-3'
	B-14	5'-TCCGCTCTGG-3'
	B-15	5'-GGAGGGTGTT-3'
	B-16	5'-TTTGCCCGGA-3'
	B-17	5'-AGGGAACGAG-3'
	B-18	5'-CCACAGCAGT-3'
	B-19	5'-ACCCCCGAAG-3'
	B-20	5'-GGACCCTTAC-3'

### Analysis of DEGs identified at two days post infiltration

**Figure 2:**
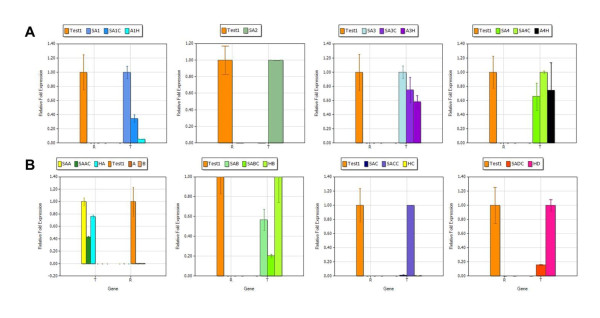
**Quantitative real time RT-PCR analysis of DEGs identified in response to βC1 at two and four days after infiltration of *N. tabacum***. SA1, SA2, SA3, SA4, SAA, SAB, SAC and SAD test samples with color indication represent the up and down regulation of differentially expressed genes as compared to calibrator having only pGreen0029 vector (SA1C, SA3C, SA4C, SAAC, SABC, SACC, SADC) and another healthy calibrator (A1H, A3H, A4H, HA, HB, HC and HD). In both (A) and (B) R stand for reference gene and T for test samples.

Differentially expressed products were cloned and sequenced. The identity of these differentially expressed genes was analysed using NCBI nucleotide data blast system. The ratio of differentially expressed genes (SA1, SA2, SA3, SA4, SAA, SAB, SAC and SAD) expressed in a sample versus a calibrator (healthy plant and plant infiltrated with pGreen0029) in comparison to a reference gene (rubisco) is indicated in the Tables 2, 3 and 4. The results of Delta Delta (Ct), Livak and the Pfaffi mathematical models indicated that SAA, SAB, SAD, SA1, SA2, and SA3 mRNA expression were upregulated in sample compared to the calibrator (plant inoculated with pGreen0029 and healthy plant). Interestingly elevation of mRNA transcripts was also detected by RT-PCR (Figure 2A and 2B). In contrast SAC and SA4 mRNA expression was down regulated in the sample compared to the calibrator (Figure 2A and 2B). The calculated expression levels by these models is indicated in the Tables 2, 3 and 4.

**Table 2:** Conclusion of relative quantification methods of differentially expressed genes at two and four days after inoculation

DEG	Length bps	Identity	Up/Down regulation
**SAA**	(287)	*S. lycopersicum *WRKY transcription factor IId-1 splice	Upregulated

**SAB**	(231)	Putative Rieske iron-sulfur protein [*A. thaliana*]Length = 539, Rieske iron-sulfur protein Tic55 [*P. sativum*]Length = 553	Upregulated

**SAC**	(386)	*N. tabacum *mitochondrial DNA, complete genome Length = 430597 NADH dehydrogenase subunit 1 NADH dehydrogenase subunit 2, 846 bp at 5' side:	Down regulated

**SAD**	(262)	Quinonprotein alcohol dehydrogenase like *M. truncatula*	Upregulated

**SA1**	(442)	Trigger factor (chaperone in protein export)	Upregulated

**SA2**	(688)	*A. thaliana *calmodulin-binding receptor-like kinase	Upregulated

**SA3**	(772)	Polyribonucleotide nucleotidyltransferase	Upregulated

**SA4**	(283)	Chromosomal replication initiator protein DnaA	Down regulated

**Table 3:** Relative quantification methods of differentially expressed genes two days post inoculation.

Genes	Identity	Relative quantification against Unit mass		Relative quantification Normalized to a reference gene		
		
		Control	Healthy	Livak method	ΔCT Method	Pfaffi Method
**SAA**	*S. lycopersicum *WRKY transcription factor IId-1 splice	2.32	1.32	0.737C/0.381H	0.942C/0.838H	0.737C/0.381H

**SAB**	Putative Rieske iron-sulfur protein [*A. thaliana*]Length = 539, Rieske iron-sulfur protein Tic55 [*P. sativum*]Length = 553	2.751	0.566	0.870C/0.162H	0.972C/0.728H	0.870C/0.162H

**SAC**	*N. tabacum *mitochondrial DNA, complete genome Length = 430597 NADH dehydrogenase subunit 1 NADH dehydrogenase subunit 2, 846 bp at 5' side	0.010	1.905	0.003C/0.547H	-0.672C/1.355H	0.003C/0.547H

**SAD**	Quinonprotein alcohol dehydrogenase like [*M. truncatula*]	5.205	0.829	1.647C/0.238H	1.167C/0.707H	1.647C/0.238H

**Table 4:** Relative quantification methods of differentially expressed genes four days post inoculation.

Gene	Identity	Relative quantification against Unit mass		Relative quantification normalized to a reference gene		
		
		Control	Healthy	Livak method	ΔCT Method	Pfaffi Method
**SA1**	Trigger factor (chaperone in protein export)	2.88	17.75	1.443C/7.727H	1.08C/1.74H	1.443C/7.727H

**SA2**	*A. thaliana *calmodulin-binding receptor-like kinase	3.759	3.759	8.564	-	8.564

**SA3**	Polyribonucleotide nucleotidyltransferase	1.32	1.70	0.664C/0.742H	0.790C/.837H	0.664C/0.742H

**SA4**	Chromosomal replication initiator protein DnaA	0.659	0.882	0.329C/0.253H	0.674C/0.706H	0.329C/0.253H

**Figure 3:**
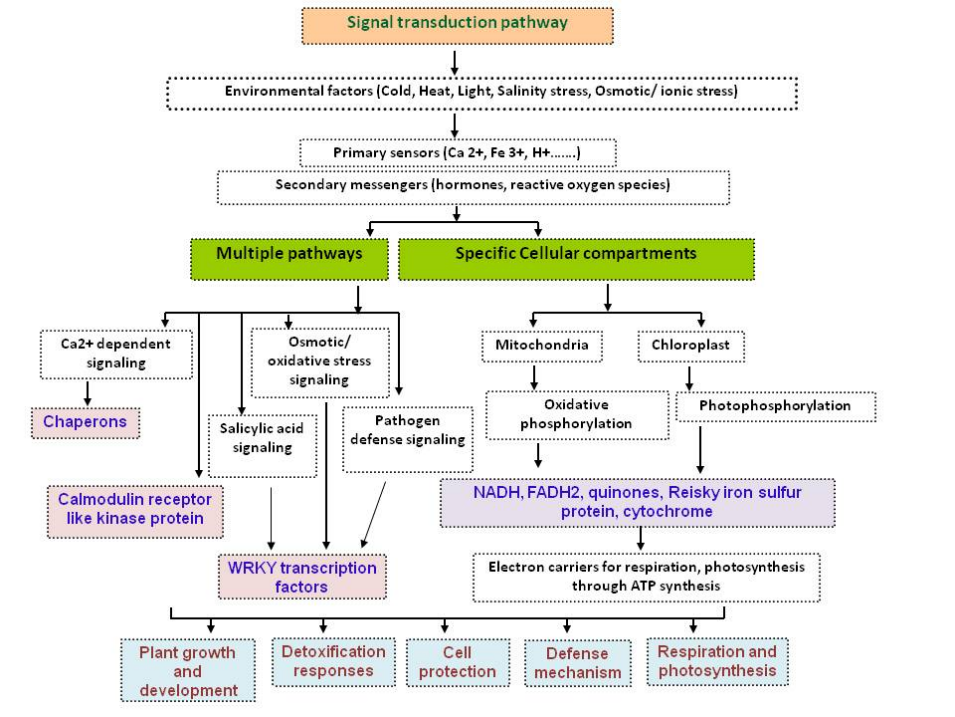
**Schematic pathway showing the involvement of the differentially expressed genes (DEGs) in signal transduction pathways**. DEGs isolated from *N. tabacum *at two and four days after infiltration in response of βC1 are involved in different pathways during host protein interactions and also segregate in specific cellular compartments. Chaperons, CRCK2 and WRKY transcription factors involved in Ca^+ ^dependent signalling, salicylic acid signalling, osmotic and oxidative stress signalling and pathogen defence signalling pathways. In contrast, NADH, Reisky iron sulphur protein and quinone protein are related to mitochondrial and chloroplast sysstems and act as electro carriers for respiration, photosynthesis by ATP synthesis. The collective role of these DEGs are in defence, cell protection, respiration, photosynthesis, detoxification, plant growth and development.

The results indicated that SAA showed 76% nucleotide sequence identity with *Solanum lycopersicum *WRKY transcription factor IId-1 splice. The results show that the SAA gene is upregulated (Figure 2) upon inoculation with the ChLCuB βC1 gene, which is a pathogenicity determinant [21-23], helps in viral movement, is involved in symptom induction [9,24,25], is a suppressor of gene silencing [26] and may be the target of a host response that up-regulates WRKY transcription factors [27]. It has been shown that the transcription of WRKY genes are strongly and rapidly upregulated in response to wounding, pathogen infection or abiotic stresses in numerous plant species, as indicated in Figure 3[28]. Infection of tobacco with *Tobacco mosaic virus *(TMV) or bacteria, or treatment with fungal elicitors, salicylic acid (SA) or H_2_O_2_, strongly induces several WRKY genes [29,30]. This suggests that the expression of βC1 gene results in a stress response and the plant responds to these stresses by increasing the transcription of WRKY genes.

SAB showed 68% nucleotide sequence identity with *A. thaliana *putative Rieske iron-sulfur protein (RISP) and 73% with *P. sativum *RISP. The expression of SAB was upregulated in the response of ChLCB βC1 (Figure 2). RISP was identified from expression of betaC1 gene [31] and is absolutely required for mitochondrial respiration (Figure 3) as reported earlier [32,33]. Mitochondrial RISP is encoded by a nuclear gene, translated as a precursor protein in the cytoplasm and post-translationally imported into mitochondria. Huang [34] demonstrated that the RISP gene family is differentially regulated; higher RISP levels occur in flowers than in leaves, stems and roots. RISP is involved in energy production in the form of ATP, required for pollen development and must be supplied mainly by mitochondria. Similarly, flower mitochondria could meet the high demand for energy either by increasing their metabolic activity to generate more ATP per mitochondrion or by increasing their number per cell so that more ATP is produced [35]. It has been shown that expression of βC1 results in foliar enations [10], which is an indication of enhanced cell division. Cell division is an energy requiring process. Therefore one possible pathway to acquire energy is via RISP proteins (Figure 3). However, this hypothesis will require further experimental confirmation.

The SAC DNA sequence shows 93% nucleotide sequence identity with both NADH dehydrogenase subunit 1 (*ndh*1) and NADH dehydrogenase subunit 2 (*ndh*2) of *N. tabacum *mitochondrial genes, also known as NADH oxidoreductase. Similar to the SAB, it has been demonstrated that SAC is a *N. tabacum *mitochondrial protein and also involved in generation of cellular energy in the form of ATP by building the electrochemical potential in electron transport chain as indicated in (Figure 3) [35,36]. The SAD transcript showed 47% nucleotide sequence identity with the *M. truncatula *quinon protein alcohol dehydrogenase. The quinon protein alcohol dehydrogenases are involved in plant development and senescence, reducing the concentration of toxic amines during stress conditions, and providing hydrogen peroxide for wall stiffening and lignification (Figure 3).

### Analysis of DEGs identified at four days post infiltration

Several genes were also identified that were differentially expressed at 4 dpi. DEG SA1 shows 99% nucleotide sequence identity with trigger factor (chaperone in protein export) of *P. acnes*. It has been suggested that molecular chaperones play a critical role in targeting proteins to the mitochondria, are involved in Ca^+ ^dependent signaling pathway (Figure 3) and in the subsequent folding of the imported protein [37-39]. It may be very useful to analyze the interaction of βC1 with chaperones through protein-protein interaction in future. It has been shown that SA2 transcript belongs to the primary calcium receptor called calmodulin (CaM; Figure 3), which is a ubiquitous protein found in both plants and animals [40]. It is located in cytoplasmic and nuclear compartments and can be attached to the plasma membrane in plant cells [41,42].

SA2 showed 72% nucleotide sequence identity with *A. thaliana *calmodulin-binding receptor-like kinase 2 (CRCK2) and, interestingly, the expression of CRCK1 is up-regulated by cold and salt stresses, as well as the stress molecules ABA (abscisic acid) and hydrogen peroxide, suggesting that CRCK2 may be involved in osmotic and oxidative stress signal transduction pathways in plants [43]. It has been suggested that CRCK2 protein is up regulated (Figure 2) during pathogen infection and also regulates the activities of a wide range of CaM binding proteins (CaMBPs), including metabolic enzymes, transcription factors such as WRKY group II d [44], ion channels, protein kinases/phosphatases and structural proteins [45,46], as indicated in Figure 3.

Transcript SA3 showed 92% nucleotide sequence identity with the polynucleotide nucleotidyltransferase from *P. cryohalolentis K5 *(PNPase; encoded by the pnp gene). PNPase is an RNA binding protein, involved in post-transcriptional gene silencing, participates in RNA degradation [47] and plays a central role in adaptation to growth at low temperature [48]. Previous studies identified PNPase in eubacteria [49-51], *Drosophila melanogaster *[52], plants [53,54], and even mice and humans [55,56]. Here it has been identified in *N. tabacum *in the response of βC1 of ChLCB. SA4 shows 90% nucleotide sequence identity with the chromosomal replication initiator protein DnaA. βC1 induces cell proliferation (enations) and a requirement for DnaA during cell division is thus consistent with this finding. Sequence analysis of the cloned DEGs showed 8 of them to represent genes that have been previously characterized (Table 5), while the remainder represent genes of unknown function and hypothetical proteins predicted from sequence. All these genes are associated with chloroplast and mitochondrial host compartments.

**Table 5:** Differentially expressed genes (DEGs) and their identities

DEG	Length of amplified fragment	Identity	**Accession No**.	Identity
		**Genes differentially expressed at two days after inoculation**		

**SAA**	287	*S. lycopersicum *WRKY transcription factor IId-1 splice	AY157059	(76%)
**SAB**	231	Putative Rieske iron-sulfur protein [*A. thaliana*]Length = 539, Rieske iron-sulfur protein Tic55 [*P. sativum*] Length = 553	NM128041 AJ000520	(68%)/(73%)
**SAC**	386	*N. tabacum *mitochondrial DNA, complete genome Length = 430597: NADH dehydrogenase subunit 1 and NADH dehydrogenase subunit 2, 846 bp at 5' side	BA000042	(93%)
**SAD**	262	Quinonprotein alcohol dehydrogenase like [*M. truncatula*]	ABE84009 ABE86610	(47%)

		**Genes differentially expressed at four days after inoculation**		

**SA1**	442	Trigger factor (Chaperone protein)	AE017283	(99%)
**SA2**	688	*A. thaliana *calmodulin-binding receptor-like kinase 2 (CRCK2)	NM116255	(72%)
**SA3**	772	Polyribonucleotide nucleotidyltransferase	CP000323	(92%)
**SA4**	263	Chromosomal replication initiator protein DnaA	CP000653	(90%)

The results suggest that the DEGs identified in response to βC1 are involved in multiple pathways; oxidative stress signaling, Ca+ dependent signaling, salicylic acid signaling pathways (Figure 3). Interestingly, these DEGs are related to specific cellular compartments, mitochondria and chloroplasts (Figure 3), where they act as electron carrier for respiration and photosynthesis by ATP synthesis (Figure 3). Collectively these genes perform their roles in plant growth and development, detoxification responses, cell protection and defense against invading viral proteins or pathogen (Figure 3).

### Analysis of DEGs using KOBAS

The DEGs responsive to ChLCB βC1 were analyzed using the KEGG orthology (KO) system, also called KOBAS (KO Based Annotation System). This showed that polyribonucleotide nucleotidyltransferase is involved in the purine and pyrimidine metabolic pathways (Table 6 and 7). These finding suggest that βC1 interact with host genes in such a manner to increase the amount of purines and pyrimidines in the cells and this is required for cell division which is induced by βC1.

**Table 6:** Result analysis of DEGs through KOBAS; KO Based Annotation System for the pathway identification

Sequence identifier	KO term	KO definition	Rank	E-value	Score	Identity (%)	Blast hit
SA3	K00962	Polyribonucleotide nucleotidyltransferase	1	1e-111	404.0	96.64	Pcr. Pcryo 0080

Each row corresponds to a query DNA or protein input by the user. The first column contains sequence identifier extracted from the input. The second column contains the assigned KO terms hyperlinked to detailed description in KEGG. The third column contains KO term definition that this protein sequence belongs to this available protein in this program. The fourth to seventh columns shows the rank, e-value, score and identity of the BLAST hit. The last column contains the gene ID of the hit hyperlinked to the KEGG GENES dataset database.

**Table 7:** Summary of purine and pyrimidine metabolic pathways of Polynucleotide nucleotidyl transferase

Query gene	Pathway	Count and ratio	p-value	q-value	Web site
SA3	Pyrimidine metabolism	**1/100% 44/1.53%**	0.0153417015342	0.023709902371	http://www.genome.jp/kegg/catalog/org_list.html http://kobas.cbi.pku.edu.cn/help.do http://kobas.cbi.pku.edu.cn http://www.genome.jp/
	
SA3	Purine metabolism	**1/100% 68/2.37%**	0.023709902371	0.023709902371	

Table 4 described the first column shows the name of the pathway. The second column lists the number and percentage of input genes or proteins involved in the pathway (top red in color) and the number and percentage of background genes or proteins involved in the pathway (bottom green in color). The third and fourth columns list the p-value and q-value of the statistical significance, respectively. Purine and pyrimidine metabolic pathways of (SA3) polynucleotide nucleotidyltransferase that is an RNA binding protein and involved in RNA degradation.

## Conclusions

From all these related results it has been concluded that the DEGs in the response of βC1 of ChLCB under 35S cauliflower promoter are related to the chloroplast and mitochondria and are involved in the ATP synthesis, act as electron carriers for respiration and photosynthesis processes. These DEGs play an important role in plant growth and development, cell protection, defence processes, replication mechanisms and detoxification responses as illustrated in Figure 3.

## Methods

### Cloning of βC1 of Chilli leaf curl betasatellite in pJIT163

The βC1 of *ChLCB *was cloned under the control of the cauliflower mosaic virus 35S promoter in the pJIT163 plant expression vector. A set of primers (ChβC135S(F) 5'-GCAAGCTTATGCACCACGTATATGAATTATGTCC-3'/ChβC135S(R) 5'- GCGAATTCTCACACACACACATTCGTACATAC-3'; having *Eco*RI and *Hin*dIII restriction sites, respectively) were designed to the reported sequence (accession no. AJ316032) to amplify a 450 bp DNA fragment containing the ChLCB βC1 gene. The fragment was amplified with an initial 94°C for 5 min followed by 30 cycles of 94°C for 1 min, 50°C for 1 min, 72°C for 1 min. A final extension at 72°C for 10 min was included. The amplification product was analyzed by 1% agarose gel electrophoresis. The amplified fragment and pJIT163 vector were restricted with *Eco*RI and *Hin*dIII restriction enzymes at 37°C overnight, precipitated with phenol-chloroform and ligated at 16°C overnight. The ligated product was transformed into *E. coli *10b. The transformation mixture was then spread on 100 mg/ml LB ampicillin petri plates after incubation for one h at 37°C. Plates were incubated overnight at 37°C and the next day colonies were cultured in LB containing ampicillin and placed overnight in a shaking water bath at 37°C. Plasmid isolation from cultures was performed by miniprep method and recombinant clone was confirmed by digestion with *Eco*RI and *Hin*dIII. The resultant recombinant clone was named pSAβC135S.

### Transfer of expression cassette to binary vector and transformation of Agrobacterium tumefaciens

pSAβC135S and pGreen0029 were restricted with *Xho*I and *Xba*I endonuclease, ethanol precipitated and ligated at 16°C for 18 h. This was used for transformation into *E. coli *and colonies were confirmed by restriction analysis. Both pGreen0029 and pGreen0029 containing the expression cassette were transformed into *Agrobacterium tumefaciens *strain (GV 3101) by electroporation. The transformation mixture was then spread on LB medium plates containing 50 μg/ml of kanamycin, 25 μg/ml of rifampicin and 100 μg/ml tetracycline antibiotics, after a one hour incubation at 28°C. Plates were incubated at 28°C until colonies appeared. After 48 hours, colonies were grown in LB liquid medium containing 50 μg/ml of kanamycin, 25 μg/ml of rifampicin and 100 μg/ml tetracycline, and placed at 28°C for 48 h. The transformants were confirmed by PCR analysis using the primers ChβC135S(F)/ChβC135S(R).

### Agroinfiltration of plants

*Agrobacterium *cultures were grown at 28°C for 48 h in liquid LB medium containing 50 μg/ml of kanamycin and 25 μg/ml of rifampicin. The bacterial cells were pelleted at 4000 rpm for 10 min at 20°C and resuspended in 10 mM MgCl_2 _and 150 μg of acetosyringone per ml. After a three hour incubation cells were infiltrated into young, fully expanded leaves of 4 week- old *N. tabacum *plants using a 5 ml syringe.

### Isolation of messenger RNA and cDNA synthesis

Infiltrated of *N. tabacum *leaves infiltrated with pGreen0029 and pGreen0029 containing the βC1 expression cassette were collected two and four days after inoculation in liquid nitrogen. Total RNA was extracted using Trizol reagent (Invitrogen, USA) following the manufacturer's instructions. The integrity and purity of total RNA isolated from infected leaf samples was assessed by electrophoresis on 1% agarose gels. The messenger RNA was isolated from total RNA using oligo (dT) cellulose columns (MRC, USA) according to the manufacturer's instructions. The loaded columns were washed with binding buffer and mRNA was eluted. The eluted mRNA was precipitated and dissolved in DEPC treated water. Messenger RNA resulting from two and four days post infiltration samples were reverse transcribed to cDNA using Revert Aid H- First Strand cDNA synthesis kit, (Fermentas, USA). Three reverse transcription reactions were carried out for each mRNA using three different anchored (T11M) primers (where M may be G, C or A). The products of reverse transcriptions (cDNA) were stored at -20°C for differential display PCR amplifications.

### Differential display analysis

PCR amplification of each cDNA (synthesized from mRNA isolated from two and four days post inoculation samples) was carried out in combination with one of the three anchored primers and one of the twenty arbitrary primers of the B-Series (as indicated in Table 1), providing 60 combinations in case of four days post inoculation and 29 combinations in the case of two days post inoculation. PCR was carried out in a final reaction volume of 50 μl containing 2.5 μl (100 ng/μl and 10 ng/μl) of first strand cDNA, 5 μl of 10× PCR buffer, 4 μl MgCl_2 _(25 mM), 1 μl of dNTPs (10 mM each), 2 μl of anchord primer (250 ng/μl), 8 μl of arbitrary primer (100 ng/μl), 0.5 μl of *Taq *DNA Polymerase (5 U/μl; Fermentas, USA), 27 μl double distilled H_2_O. The PCR amplification protocol included first cycle at 94°C for 4 min followed by 45 cycles of 36°C for 2 min, 72°C for 1 min, 94°C for 1 min; and a final extension step at 72°C for 10 min. The amplified PCR products were resolved on 1% agarose gel and stained with ethidium bromide.

### Cloning and sequencing of differentially expressed genes (DEGs)

The differentially expressed bands were excised from the gel and extracted by QIAGEN gel extraction kit and DNA extraction kit (MBI, Fermentas). The eluted bands were ligated into pTZ57RT, and transformed into *E. coli *TOP 10 by the heat shocked method. Plasmid DNA was isolated using the miniprep method and clones were confirmed by restriction analysis using *Eco*RI and *Pst*I restriction enzymes. Purified clones were sequenced using M13 (-20) forward and M13 (-26) reverse primers and BigDye terminator v 3.1 ABI Prism 310 Genetic analyzer (Applied Biosystems, USA) as decribed by the manufacturer. Sequence information was stored, assembled and analysed using the Lasergene sequence analysis package (DNAStar Inc., Madison, WI, USA) running on an IBM compatible PC.

### Analysis of DEGs using NCBI, KOBAS and RT-PCR

The nucleotide sequences were analyzed using BLAST; for blastn and blastx algorithms in NCBI. Clusters of orthologus group of proteins were identified at NCBI http://www.ncbi.nlm.nih.gov/Blast.cgi and KEGG orthology http://www.genome.jp/. Real time quantitative PCR was performed to analyse expression of DEGs in relation to a reference gene and the calibrators at a constant level of fluorescence. These were calculated with Delta Delta (Ct), Livak and the Pfaffi mathematical models of quantitative real time PCR method [57,58]. For RT-PCR each sample was used in triplicate and the experiment was repeated three times to confirm the reproducibility of result. The sequences of RT-PCR primers are shown in Table 8 and 9.

**Table 8:** After two days differentially expressed genes (DEGs) primer sequences for quantitative real time PCR

Name of genes	Sequences of primers	MERS
**Primer sequences two days post inoculation of βC1 of ChLCB for Q-RTPCR analysis**		

**SAA**	SAA F: GAGACCCGGGATGTCCTGGCAAGAAAGCAT	**(30 MERS)**
	SAAQPCR:AATTACAAAAGAGCCCCTAAATCCCTAAGC	**(30 MERS)**
		
	SAA F2: GGAGAGGGCAACCGATGA	**(18 MERS)**
	SAA QPCR2: CCCCTAAATCCCTAAGC	**(17 MERS)**
		
	SAA F3: GGGACGATCGCCGGCGCCGG	**(20 MERS)**
	SAA QPCR3: TCACTACCCACCGTATC	**(17 MERS)**

**SAB**	SAB F: AATCCCCGGGATGTATGCTCCGAATCCCGC	**(30 MERS)**
	SABQPCR:CATAGTGATGTCGAAAGCAAAAGTAGGGCC	**(30 MERS)**
		
	SAB F2: GTATGCTCCGAATCCCG	**(17 MERS)**
	SAB QPCR2: CAAAAGTAGGGCCTTCC	**(17 MERS)**
		
	SAB F3: CCAGCTAAGGGAGGAATC	**(18 MERS)**
	SAB QPCR3: GGCCTTCCACTGTCTTCCTG	**(20 MERS)**

**SAC**	SAC F: TCCCCCCGGGATGTTTCAGGTTCACATGAA	**(30 MERS)**
	SACQPCR:TAGGCTATAGGTGGGGGACAATGTAGACTG	**(30 MERS)**
		
	SAC F2: CACAACACGACTCCCTAC	**(18 MERS)**
	SAC QPCR2: GAAGTTGGGCCCACCTG	**(17 MERS)**
		
	SAC F3: CTCCACGAGTCTTCATCCCC	**(20 MERS)**
	SAC QPCR3: CCGAGATCGAGAGCTTTC	**(18 MERS)**

**SAD**	SA D F; GGTGCCCGGGATGGCAGATCAGTGGAGTTG	**(30 MERS)**
	SADQPCR: GATTAGGTTCCCGTAGATAGATGCATAACC	**(30 MERS)**
		
	SAD F2: AAGTTCTAATTCGGAGGG	**(18 MERS)**
	SAD QPCR2: TAGATAGATGCATAA	**(17 MERS)**
		
	SAD F3: GTTAGCTTACTTAAACAG	**(20 MERS)**
	SAD QPCR3: TAGATGCATAACC	**(17 MERS)**

**Table 9:** After four days differentially expressed genes (DEGs) primer sequences for quantitative real time PCR

Name of genes	Sequences of primers	MERS
**Primer sequences four days post inoculation of βC1 of ChLCB for Q-RTPCR analysis**		

**SA1**	SA1 F: GTCACCCGGGATGTGACGCCGACGGTCAAT	**(30 MERS)**
	SA1 QPCR: GGGCCGCACCATGGTCCTGCTGACTTACCG	**(30 MERS)**
		
	SA1 F2: GACGGTCAATCCATGTAT	**(18 MERS)**
	SA1 QPCR2: GGTGTCAGGAGACCCCTTCCA	**(17 MERS)**
		
	SA1 F3: GGTAGAGCCCCAGTCTTCCA	**(20 MERS)**
	SA1 QPCR3: GCACCCGCCCAACTCCACGG	**(17 MERS)**

**SA2**	SA2 F: AATACCCGGGATGATAAACATTTGGGGG	**(30 MERS)**
	SA2 QPCR: CCAATGTCTAGTCTTGATGCAAAATCAA	**(30 MERS)**
		
	SA2 F2: CTAGTAAAGTTTTATGGATTCTTGGA	**(17 MERS)**
	SA2 QPCR2: ATGGATAATAGGGTGATCAGT	**(17 MERS)**
		
	SA2 F3: CACTTGGACTGTGGTCCTG	**(18 MERS)**
	SA2 QPCR3: GTCAGCCACCTTAGCTCG	**(20 MERS)**

**SA3**	SA3 F: CGCGCCCGGGATGCATCTAGATTGTCCACA	**(30 MERS)**
	SA3 QPCR: TCAATCAGACGCGAGGTTAAGGTTTCAGAC	**(30 MERS)**
		
	SA3 F2: GAAGGCTATGTAAACGAG	**(18 MERS)**
	SA3 QPCR2: GCTCTTCAAGGGTCGGGTTCAG	**(17 MERS)**
		
	SA3 F3: GACTTGGTCGTCGCTGGTA	**(20 MERS)**
	SA3 QPCR3: GCTTGATCGCGTACAGG	**(18 MERS)**

**SA4**	SA4 F: GCGACCCGGGAtGCATCTAGATTTGGGGGA	**(30 MERS)**
	SA4 QPCR: AGAAACAGAAGATCTCTGGCTCAGTTTAGG	**(30 MERS)**
		
	SA4 F2: TTCATGATTGTTGGCGCAC	**(18 MERS)**
	SA4 QPCR2: CTGATCTTCCTGTGGA	**(17 MERS)**
		
	SA4 F3: CGGCATGACCCTGTGTAA	**(20 MERS)**
	SA4 QPCR: GGGGGACTCGCGCCAGG	**(17 MERS)**

## Competing interests

The authors declare that they have no competing interests.

## Authors' contributions

SA conducted all the experimental work and drafted the manuscript. AB and IA helped in the RT-PCR and DD-PCR analysis. SM and RWB together designed the experiments. IA and SM had proof-read and finalized the manuscript. All authors read and approved the final manuscript.

## References

[B1] Hanley-Bowdoin L Settlage SB Orozco BM Nagar S Robertson D Geminviruses: models for plant DNA replication, transcription, and cell cycle regulation Crit Rev Plant Sci 1999 18 71 106 10.1016/S0735-2689(99)00383-4 10821479

[B2] Laufs J Traut W Heyraud F Matzeit V Rogers SG Schell J Gronenborn B *In vitro *cleavage and joining at the viral origin of replication by the replication initiator protein of tomato yellow leaf curl virus Proc Natl Acad Sci USA 1995 92 3879 3883 10.1073/pnas.92.9.3879 7732000 PMC42065

[B3] Mansoor S Briddon RW Zafar Y Stanley J Geminivirus disease complexes: an emerging threat Trends Plant Sci 2003 8 128 134 10.1016/S1360-1385(03)00007-4 12663223

[B4] Saunders K Bedford ID Yahara T Stanley J The earliest recorded plant virus disease Nature 2003 422 831 10.1038/422831a 12712190

[B5] Saeed M Behjatnia SA Mansoor S Zafar Y Hasnain S Rezaian MA A single complementary-sense transcript of a geminiviral DNA β satellite is determinant of pathogenicity Mol Plant Microbe Interact 2005 18 7 14 10.1094/MPMI-18-0007 15672813

[B6] Saunders K Bedford ID Briddon RW Markham PG Wong SM Stanley J A unique virus complex causes Ageratum yellow vein disease Proc Natl Acad Sci USA 2000 97 6890 6895 10.1073/pnas.97.12.6890 10841581 PMC18771

[B7] Zhou X Xie Y Peng Y Zhang Z *Malvastrum yellow vein virus*, a new *Begomovirus *species associated with satellite DNA molecule Chi Sci Bull 2003 48 2205 2209 10.1360/03wc0272

[B8] Hussain M Mansoor S Amin I Iram S Zafar Y Malik KA Briddon RW First report of cotton leaf curl disease affecting chili peppers Plant Pathol 2003 56 906

[B9] Briddon RW Bull SE Amin I Idris AM Mansoor S Bedford ID Dhawan P Rishi N Siwatch SS Abdel-Salam AM Brown JK Zafar Y Markham PG Diversity of DNA β, a satellite molecule associated with some monopartite begomoviruses Virology 2003 312 106 121 10.1016/S0042-6822(03)00200-9 12890625

[B10] Qazi J Amin I Mansoor S Iqbal MJ Briddon RW Contribution of the satellite encoded gene βC1 to cotton leaf curl disease symptoms Virus Res 2007 128 135 139 10.1016/j.virusres.2007.04.002 17482706

[B11] Soosaar JL Burch-Smith TM Dinesh-Kumar SP Mechanisms of plant resistance to viruses Nat Rev Microbiol 2005 3 789 798 10.1038/nrmicro1239 16132037

[B12] Huang Z Yeakley JM Garcia EW Holdridge JD Fan JB Whitham SA Salicylic acid-dependent expression of host genes in compatible *Arabidopsis*-virus interactions Plant Physiol 2005 137 1147 1149 10.1104/pp.104.056028 15728340 PMC1065414

[B13] Sui C Fan Z Wong SM Li H Cloning of cDNAs encoding the three subunits of oxygen evolving complex in *Nicotiana benthamiana *and gene expression changes in tobacco leaves infected with *Tobacco mosaic virus* Physiol Mol Plant Pathol 2006 68 61 68 10.1016/j.pmpp.2006.06.003

[B14] Havelda Z Maule AJ Complex spatial responses to cucumber mosaic virus infection in susceptible *Cucurbita pepo *cotyledons Plant Cell 2000 12 1975 1986 10.1105/tpc.12.10.1975 11041891 PMC149134

[B15] Kang BC Yeam I Jahn MM Genetics of plant virus resistance Annu Rev Phyto 2005 43 581 621 10.1146/annurev.phyto.43.011205.141140 16078896

[B16] Liang P Pardee AB Differential display of eukaryotic messenger RNA by means of the polymerase chain reaction Science 1992 257 967 971 10.1126/science.1354393 1354393

[B17] Yun-Jee KIM Chae-ll KWAK Young-Yun GU In-Taek H Chun Jong-Yoon Annealing control primer system for identification of differentially expressed genes on agarose gels BioTechniques 2004 36 424 434 15038158 10.2144/04363ST02

[B18] Crauwels M Winderickx J de Winde JH Thevelein JM Identification of genes with nutrient-controlled expression by PCR-mapping in the yeast *Saccharomyces cerevisiae.* Yeast 1997 13 973 984 10.1002/(SICI)1097-0061(199708)13:10<973::AID-YEA146>3.0.CO;2-S 9271111

[B19] Gross C Watson K Application of mRNA differential display to investigate gene expression in thermotolerant cells of *Saccharomyces cerevisiae.* Yeast 1998 14 431 442 10.1002/(SICI)1097-0061(19980330)14:5<431::AID-YEA242>3.0.CO;2-V 9559551

[B20] Tawe WN Eschbach ML Walter RD Henkle-Duhrsen K Identification of stress-responsive genes in Caenorhabditis elegans using RT-PCR differential display Nucleic Acids Res 1998 26 1621 1627 10.1093/nar/26.7.1621 9512531 PMC147444

[B21] Cui X Li G Wang D Hu D Zhou X A begomovirus DNA β encoded protein binds DNA, functions as a suppressor of RNA silencing, and targets the cell nucleus J Virol 2005 79 10764 10775 10.1128/JVI.79.16.10764-10775.2005 16051868 PMC1182626

[B22] Gopal P Pravin Kumar P Sinilal B Jose J Kasin Yadunandam A Usha R Differential roles of C4 and βC1 in mediating suppression of post-transcriptional gene silencing: evidence for transactivation by the C2 of Bhendi yellow vein mosaic virus, a monopartite begomovirus Virus Res 2007 123 9 18 10.1016/j.virusres.2006.07.014 16949698

[B23] Saunders K Norman A Gucciardo S Stanley J The DNA β satellite component associated with ageratum yellow vein disease encodes an essential pathogenicity protein (βC1) Virology 2004 324 37 47 10.1016/j.virol.2004.03.018 15183051

[B24] Briddon RW Mansoor S Bedford ID Pinner MS Saunders K Stanley J Zafar Y Malik KA Markham PG Identification of DNA components required for induction of cotton leaf curl disease Virology 2001 285 234 243 10.1006/viro.2001.0949 11437658

[B25] Jose J Usha R Bhendi yellow vein mosaic disease in India is caused by association of a DNA β satellite with a begomovirus Virology 2003 305 310 317 10.1006/viro.2002.1768 12573576

[B26] Sharma P Matsuda N Bajet NB Ikegami M Molecular analysis of new isolates of Tomato leaf curl Philippines virus and an associated betasatellite occurring in the Philippines Arch Virol 2010 DOI: 10.1007/s00705-010-0837-3 21053032 10.1007/s00705-010-0837-3

[B27] Zhang Y Fan W Kinkema M Li X Dong X Interaction of NPR1 with basic leucine zipper protein transcription factors that bind sequences required for salicylic acid induction of the *PR-1 *gene Proc Natl Acad Sci USA 1999 96 6523 6528 10.1073/pnas.96.11.6523 10339621 PMC26915

[B28] Eulgem T Rushton PJ Robatzek S Somssich IE The WRKY superfamily of plant transcription factors Trends Plant Sci 2000 5 199 206 10.1016/S1360-1385(00)01600-9 10785665

[B29] Chen C Chen Z Isolation and characterization of two pathogen- and salicylic acid-induced genes encoding WRKY DNA-binding proteins from tobacco Plant Mol Biol 2000 42 387 396 10.1023/A:1006399311615 10794538

[B30] Vandenabeele S Van der Kelen K Dat J Gadjev I Boonefaes T Morsa S Rottiers P Slooten L Van Montagu M Zabeau M A comprehensive analysis of hydrogen peroxide-induced gene expression in tobacco Proc Natl Acad Sci USA 2003 100 16113 16118 10.1073/pnas.2136610100 14671332 PMC307701

[B31] Rieske JS Zaugg WS Hansen RE Studies on the electron transfer system. LIX. Distribution of iron and of the component giving an electron paramagnetic resonance signal at g = 1.90 in subfractions of complex 111 J Biol Chem 1964 239 3023 3030 14217891

[B32] Beckmann JD Ljungdahl PO Trumpower BL Mutational analysis of the mitochondrial Rieske iron-sulfur protein of *Sacchammyces cerevisiae*. 1. Construction of a RlPl deletion strain and isolation of temperature-sensitive mutants J Biol Chem 1989 264 3713 3722 2645276

[B33] Trumpower BL Edwards CA Purification of a reconstitutively active iron-sulfur protein (oxidation-factor) from succinate-cytochrome c reductase complex of bovine heart mitochondria J Biol Chem 1979 254 8697 8706 224062

[B34] Huang J Struck F Matzinger DF Levings CS Functional analysis in yeast of cDNA coding for the mitochondrial Rieske iron-sulfur protein of higher plants Proc Natl Acad Sci USA 1991 88 10716 10720 10.1073/pnas.88.23.10716 1961737 PMC53001

[B35] Burrows PA Sazanov LA Svab Z Maliga P Nixon PJ Identification of a functional respiratory complex in chloroplasts through analysis of tobacco mutants containing disrupted plastid *ndh *genes EMBO J 1998 17 868 876 10.1093/emboj/17.4.868 9463365 PMC1170436

[B36] Sazanov LA Burrows PA Nixon PJ The plastid *ndh *gene code for an NADH-specific dehydrogenase: Isolation of a complex I analogue from peathylakoid membranes Proc Natl Acad Sci USA 1998 95 1319 1324 10.1073/pnas.95.3.1319 9448329 PMC18756

[B37] Horst M Oppliger W Rospert S Schonfeld HJ Schatz G Azem A Sequential action of two Hsp70 complexes during protein import into mitochondria EMBO J 1997 16 1842 1849 10.1093/emboj/16.8.1842 9155010 PMC1169787

[B38] Neupert W Protein import into mitochondria Annu Rev Biochem 1997 66 863 917 10.1146/annurev.biochem.66.1.863 9242927

[B39] Ryan MT Naylor DJ Hoj PB Clark MS Hoogenraad NJ The role of molecular chaperones in mitochondrial protein import and folding Int Rev Cytol 1997 174 127 193 10.1016/S0074-7696(08)62117-8 9161007

[B40] Zielinski RE Calmodulin and calmodulin binding proteins in plants Annu Rev Plant Physiol Plant Mol Biol 1998 46 697 725 10.1146/annurev.arplant.49.1.697 15012251

[B41] White PJ Broadley MR Calcium in plants Ann Bot 2003 92 487 511 10.1093/aob/mcg164 12933363 PMC4243668

[B42] Trewavas A Signal perception and transduction. In BB Buchanan, W Gruissem, RL Jones, eds, Biochemistry and molecular biology of plants American Society of Plant Biologists Rockville 2000 1 962 973

[B43] Yang T Chaudhuri S Yang L Chen Y Poovaiah BW Calcium/calmodulin up-regulates a cytoplasmic receptor-like kinase in plants J Biol Chem 2004 279 42552 42559 10.1074/jbc.M402830200 15292241

[B44] Chan YP Lee JH Yoo JH Moo BC Choi MS Kang YH Lee SM Kim HS Kang KY Chung WS Lim CO Cho MJ WRKY group IId transcription factors interact with calmodulin FEBS Lett 2005 579 1545 1550 10.1016/j.febslet.2005.01.057 15733871

[B45] Snedden W Fromm H Calmodulin as a versatile calcium signal transducer in plants New Physiologist 2001 151 35 66 10.1046/j.1469-8137.2001.00154.x 33873389

[B46] Hoeflich KP Ikuru M Calmodulin in action: diversity in target recognition and activation mechanisms Cell 2002 108 739 742 10.1016/S0092-8674(02)00682-7 11955428

[B47] Bernstein JA Lin PH Cohen SN Lin-Chao S Global analysis of *Escherichia coli *RNA degradosome function using DNA microarrays Proc Natl Acad Sci USA 2004 101 2758 2763 10.1073/pnas.0308747101 14981237 PMC365694

[B48] Yamanaka K Inouye M Selective mRNA degradation by polynucleotide phosphorylase in cold shock adaptation in *Escherichia coli* J Bacteriol 2001 183 2808 2816 10.1128/JB.183.9.2808-2816.2001 11292800 PMC99497

[B49] Favaro R Deho G Polynucleotide phosphorylase-deficient mutants of *Pseudomonas putida* J Bacteriol 2003 185 5279 5286 10.1128/JB.185.17.5279-5286.2003 12923102 PMC180990

[B50] Goverde RL Huisin't Veld JH Kusters JG Mooi FR The psychrotrophic bacterium Yersinia enterocolitica requires expression of pnp, the gene for polynucleotide phosphorylase, for growth at low temperature (5°C) Mol Microbiol 1998 28 555 569 10.1046/j.1365-2958.1998.00816.x 9632258

[B51] Symmons MF Jones GH Luisi BF A duplicated fold is the structural basis for polynucleotide phosphorylase catalytic activity, processivity, and regulation Structure Fold Des 2000 8 1215 1226 10.1016/S0969-2126(00)00521-9 11080643

[B52] Leszczyniecka M DeSalle R Kang DC Fisher PB The origin of polynucleotide phosphorylase domains Mol Phylogenet Evol 2004 31 123 130 10.1016/j.ympev.2003.07.012 15019613

[B53] Kudla J Hayes R Gruissem W Polyadenylation accelerates degradation of chloroplast mRNA EMBO J 1996 15 7137 7146 9003789 PMC452540

[B54] Yehudai-Resheff S Hirsh M Schuster G Polynucleotide phosphorylase functions as both an exonuclease and a poly(A) polymerase in spinach chloroplasts Mol Cell Biol 2001 21 5408 5416 10.1128/MCB.21.16.5408-5416.2001 11463823 PMC87263

[B55] Leszczyniecka M Kang DC Sarkar D Su ZZ Holmes M Valerie K Fisher PB Identification and cloning of human polynucleotide phosphorylase, hPNPase old-35, in the context of terminal differentiation and cellular senescence Proc Natl Acad Sci USA 2002 99 16636 16641 10.1073/pnas.252643699 12473748 PMC139196

[B56] Sarkar D Leszczyniecka M Kang DC Lebedeva IV Valerie K Dhar K Pandita S Fisher PB Down-regulation of Myc as a potential target for growth arrest induced by human polynucleotide phosphorylase (hPNPaseold-35) in human melanoma cells J Biol Chem 2003 278 24542 24551 10.1074/jbc.M302421200 12721301

[B57] Pfaffi MW Hageleit M Validities of mRNA quantification using recombinant RNA and recombinant DNA external calibration curves in real time RT-PCR Biotechnol Lett 2001 23 275 282 10.1023/A:1005658330108

[B58] Michael WP A new mathematical model for relative quantification in real time RT-PCR Nucleic Acids Res 2001 29 2001 2007 10.1093/nar/29.9.e45 PMC55695 11328886

